# Seroprevalence of brucellosis and associated risk factors among abattoir workers in Bauchi State, Nigeria

**DOI:** 10.11604/pamj.2020.35.33.18134

**Published:** 2020-02-07

**Authors:** Philip Bobu Igawe, Emmanuel Okolocha, Grace Sabo Kia, Istifanus Bugun Irmiya, Muhammad Shakir Balogun, Patrick Nguku

**Affiliations:** 1 Nigeria Field Epidemiology and Laboratory Training Programme (NFELTP), Abuja, Nigeria; 2 Department of Veterinary Public Health and Preventive Medicine, Ahmadu Bello University, Zaria, Nigeria; 3 Department of Epidemiology, Bauchi State Ministry of Agriculture and Natural resources, Bauchi, Nigeria

**Keywords:** Brucellosis, seroprevalence, abattoir, occupational hazard, ELISA, Nigeria

## Abstract

**Introduction:**

Brucellosis is a reemerging and neglected zoonotic disease. It is an occupational bio-hazard and a public health problem. The objective of the study was to determine the seroprevalence of brucellosis and its risk factors among abattoir workers in Bauchi state.

**Methods:**

A cross-sectional study was conducted in the three senatorial district abattoirs of Bauchi State. Abattoir workers (n=284) were selected by stratified random sampling. Data were collected using an adapted questionnaire. Serum samples collected, were screened for brucellosis with Rose Bengal Plate Test (RBPT), tested with Enzyme Linked Immunosorbent Assay (ELISA). Seropositive participants were positive for both RBPT and ELISA. Data were described in proportions and analyzed using bivariate and multivariate analysis.

**Results:**

Participants were all male, age range: 18-70 years (mean 35 ±13 years). Ninety-five participants were seropositive (seroprevalence 33.5%) after laboratory testing. Following bivariate analysis, using personal protective equipment (PPE) [OR: 0.5 CI95%=0.3>OR: 0.5 CI95%=0.3-0.9] was significantly protective against brucellosis. Slaughtering of animals (OR: 2.19 CI95%= 1.2-3.7), assisting in animal parturition (OR: 2.25 CI95%= 1.3-3.7), working with an open cut/wound (OR:2.1 CI95%= 1.1-3.9) and eating while working in the abattoir [OR:2.4 CI95%= 1.1>OR:2.4 CI95%= 1.1-4.9] were risks of brucellosis. Multivariate analysis showed that slaughtering of animals: Adjusted Odds-Ratio (AOR) = 1.92; CI95% = 1.03 - 3.59) and assisting in animal parturition (AOR = 2.43; CI95% = 1.40 - 4.23) remained significantly associated with brucellosis.

**Conclusion:**

Seroprevalence of brucellosis among abattoir workers in Bauchi state is high. Workers should use PPEs and animal parturitions should be handled by trained personnel alone.

## Introduction

Brucellosis is one of the most common and important global zoonotic disease, [1,2] with reported worldwide incidence of human brucellosis at >200 per 100,000 population [3]. It is caused by a fastidious, intracellular, non-spore forming, non-motile, non-encapsulated, gram negative coccobacillus bacterium of the genus *Brucella* [4]. Brucellosis is listed by the World Health Organization (WHO) as a “neglected” zoonotic. The disease has a great impact on animal and human health as well as socio-economic impact in developing countries [5]. Due to abortion, lower milk production and reduced fertility in livestock and serious health problem in human beings [6]. Animals are the natural hosts of the *Brucella* organisms and are reservoirs for human infection. Brucellosis incidents have been reported in terrestrial and marine mammals also in domestic animals [7]. Humans acquire the infection through contaminated environments/tissue, foodborne transmissions, inhalation. Occupational exposure usually results from direct contact with infected animals [6] and animal product: blood, placenta or uterine secretions of infected animals, the bacteria gains access through breaks in the skin and mucosa [5,6]. This involves abattoir workers, farm laborer, animal keepers, butchers, veterinarians and laboratory workers, where *Brucella* melitensis and *B suis* species are more virulent for humans than *B abortus* and *B canis* [6,8].

Brucellosis in humans is a systemic disease characterized by sever/acute, insidious onset of continued, intermittent, undulant or irregular fever of variable duration, headache, profuse sweating, chills, weakness, generalized aching and joint pain [9]. Relapses hypersensitivity reactions [10] are common; focal lesions occur in bones, joints, genitourinary tract and other sites. Its clinical picture is not specific in animals or humans and diagnosis needs to be supported by laboratory tests. A history of recent exposure to a known or probable source of *Brucella spp*, occupational exposure or residence in a high infection prevalence is a probable case of brucellosis. Differential diagnosis can be achieved by demonstration of by a validated serological method of *Brucella* antigen in blood (seroprevalence) or other tissue sample [6]. Brucellosis is endemic in Nigeria, resulting in massive economic losses of man-hours in infected people [11]. There are major gaps in epidemiological data, diagnostics, surveillance and control. Information essential for evaluation of zoonotic potential and for establishment of control measures is still lacking [12]. Brucellosis was found to be endemic among cattle in the three senatorial zones of Bauchi state Nigeria [13]. This study aimed to determine the seroprevalence and exposure factors associated with human brucellosis among abattoir workers in Bauchi State, Nigeria, so that policymakers and stakeholders know the extent and factors associated with the problem and can make informed decisions in the control of brucellosis in Nigeria.

## Methods

**Study sites:** the major abattoirs were selected from each of the three senatorial districts of Bauchi state, namely: the Bauchi main abattoir, Inkil, Gombe road, Bauchi LGA (Bauchi South Senatorial District); the Misau abattoir, Gamawa road, Misau LGA (Bauchi Central Senatorial District); the Azare abattoir, opposite cattle market, Kano road, Azare, Katagum LGA (Bauchi North Senatorial District). All the abattoirs are Government owned and managed by the Bauchi State Ministry of Agriculture, Bauchi, Bauchi State.

**Study design:** the cross-sectional study was carried out in the selected abattoirs following the selection criteria to determine the seroprevalence of brucellosis among abattoir workers in Bauchi State.

**Study population:** the study population was made up of abattoir workers at the three selected abattoirs in Bauchi State.

**Inclusion criteria:** all abattoir workers actively participating in abattoir operations, who were 18 years and above and present at the abattoir at the time of visit were included in the study. A seropositive individual is an abattoir worker who having been screened for *Brucella abortus* or *Brucella melitensis* antibodies had a positive test result for Rose Bengal Plate Test (RBPT) and Enzyme Linked Immunosorbent Assay (ELISA). A seronegative individual was any person working in the same abattoir whose serum was collected at the same time with the seropositive individual and who on screening with the same methods as the seropositive individual, had a negative serological test result.

**Exclusion criteria:** individuals under 18 years of age and meat buyers at the abattoir at the time of visit that are not abattoir workers were excluded from the study.

**Sampling method and recruitment:** a stratified sampling technique was adopted for the selection of participants. The abattoir workers were divided into six groups based on the types of work they perform at the abattoirs: slaughterers, meat sellers, slaughterers/meat sellers, livestock farmers/sellers, meat inspectors and others. Proportionate allocation was used to determine the number of workers to be selected from each group. Sampling frames from the different groups were prepared and from each frame, individuals were selected by simple random sampling. Number of subjects and sample size: the sample size of 284 was calculated using the formula:

$$\text{n}=\frac{\left(Z\alpha )2PX(1-P\right)}{d^{2}}$$

[14]. Using the above formula, where Z = 1.96, P = 0.24 (2013 Abuja study) [15], q = 1 - p = 0.76 and d = 0.05 Since the total population (N) 3026 is less than 10,000 and sample (n) is greater than 5% of the total population, we then calculate the final population (nf) using the Finite Population Correction (FPC) formula:

$$nf=\frac{n}{1+(n-1)/N}$$

where N=3026, n=280 280/ (1+280/3026) =256 Finite population correction factor Allowing for an anticipated Non response of 10%, the sample size becomes:

$$\text{N}=\frac{n}{\left(1-nr\right)}$$

256/ (1-0.1) = 284 Method of allocation to study groups: the sample size of 284 was calculated earlier, using a proportion of 0.7 the workers were selected from the various work groups. There were 539 workers working at the abattoirs at the time of study out of which there were: slaughterers 92/131 (Workers involved in preparing the animals for slaughter, slaughtering, cleaning the carcass and preparing it to be sold to meat sellers stalls in quarters); meat sellers 80/114 (workers involved in selling meat cuttings in weights to walking customers and households from their market stalls or tables), slaughterers/meat sellers 73/104 (workers that function as both slaughterers and meat sellers); meat inspectors 15/21 (staff of the state and local government authorities responsible for meat hygiene and inspection at the abattoirs); livestock farmers/ sellers 11/15 (farmers and animal dealers who bring their livestock to the abattoir for onward sale slaughterers, meat sellers of other dealers); others 14/20 (including: meat transporters, cleaners, security men, revenue/admin officer, poultry butchers).

**Data collection:** the study was conducted between January and September 2017. Data was collected through interviewer-administered questionnaires by trained personnel. Qualified medical personnel collected about 3ml. of blood under strict hygienic conditions using sterile syringes and needles from a vein in the upper arm of individual abattoir workers into 5ml plain serum tubes and kept in slanted position on ice. Sera were collected in sterile vials, each vial containing serum sample was labelled with a code that matched it to the study site and study participant. The codes and identification details were recorded safely on an MS Excel sheet document. The samples were stored at -20^o^C until transported for analysis at the laboratory.

**Laboratory analysis:** serological testing of abattoir workers: each sample was screened (in parallel) for *Brucella abortus* and *Brucella melitensis* antibodies using RBPT specific for each antigen and ELISA was carried out using human IgG and IgM ELISA kits specific for *B. abortus* outer membrane. The screening and testing of the serum samples were carried out at the Department of Veterinary Public Health Laboratory ABU. Zaria. *Brucella melitensis* and *Brucella abortus* antigens for Rose Bengal Plate Test (RBPT) were sourced from the veterinary laboratory agency, Weybridge, United Kingdom. *Brucella* IgG and IgM ELISA kits using *Brucella abortus* outer membrane (which is shared by other species), were sourced from Calbiotech Inc. Spring Valley, California, USA. The tests were done using the standard protocol available in the 2009 Terrestrial Manual [16]. We tested for *Brucella abortus* antibodies using RBPT specific for *Brucella abortus* and *Brucella melitensis*. Serum samples and antigen were brought to room temperature (22 ± 4^°^C). Between 25-30µl of each serum was placed on a white tile and an equal volume of antigen was placed near each serum spot. They were mixed thoroughly with a clean wooden rod and after 4-minutes read for agglutination. The agglutination reactions were recorded as positive (+) or negative (-). The observance of agglutination gave a positive test result while absence of visible agglutination was recorded as negative for agglutination. The ELISA test was performed on test samples screened positive for agglutination. The reagents in the kit were reconstituted and the test procedure was carried out according to manufacturers´ instructions. Participants were considered as positive based on a positive RBPT and ELISA result.

**Statistical analyses:** data obtained was entered into Epi info version 7.1.3.10. It was summarized using descriptive and analytic statistics. MS Excel was used to generate tables, graphs and charts. Differences in seronegatives and seropositives were compared using Odds Ratios and Chi square tests. Test results were considered as significant if the Chi-square test p-value was < 0.05. Factors. A multivariate analysis (logistic regression) on significant factors from the bivariate analysis was carried out. By stepwise elimination we determined factors that remained significantly associated with a positive serological test for brucellosis.

**Ethical considerations:** approval for this study was obtained from the Bauchi state research ethics committee. Permission was sought for and obtained from the management of each abattoir where the study was carried out through the state Ministry of Agriculture, Bauchi and Informed consent was obtained from each eligible abattoir worker before questionnaire administration and sample collection.

## Results

Participants in this study numbered 284. Data were collected from the Bauchi, Misau and Azare abattoirs. The results are presented below in tables and charts in line with the specific objectives of the study in terms of the socio-demographic characteristics of the respondents. All participants were male (100%), with an age ranged between 18 to 70 years (mean age 35±13 years). Most (49.6%) of the samples collected at the Bauchi LGA abattoir. About half of the participants 133 (46.8%) had primary school education. There were 38 (13.4%) participants with tertiary level education, they were the least, while 64 (22.5%) had had no formal education. The majority 96 (33.8%) of the abattoir workers were within the age range 21-30 years. Most of the abattoir workers were married 208 (73.2%). Thirty six percent (36%) of the participants indicated more than 20 years of employment at the abattoir with fewer participants working for less than one year (0.4%). Most of the abattoir workers were involved in slaughtering of the live animals, processing the carcasses and also selling the meat/carcasses at the market stalls i.e. the slaughterer/meat seller group 98 (34.5%) (Table 1). All (284) samples were screened for brucellosis using the Rose Bengal Plate Test (RBPT). Ninety-eight [98 (34.5%)] of the samples had positive screening results. These positive samples were subjected to further assay using *Brucella* IgG and IgM ELISA kits specific for *Brucella* outer membrane. Seropositivity following ELISA found 18 (18.9%) positive with human IgG alone and 6 (6.1%) samples were positive with human IgM alone. While 71 (74.15%) samples were reactive to both IgG and IgM, giving overall seroprevalence of 33.5% (Table 2).

**Table 1:** Socio-demographic characteristics of abattoir workers screened for brucellosis in Bauchi state

Characteristics	Total sampled	Proportion Sampled (%)	Seropositive Workers N(95)	Seropositive Workers (%)	Seronegative Workers N(189)	Seronegative Workers (%)
**Abattoirs**						
Azare Abattoir	63	22.2	38	60	25	40
Bauchi Abattoir	141	49.6	35	24.8	106	75.2
Misau Abattoir	80	28.5	22	27.5	58	72.5
**Sex**						
Female	0	0	0	0	0	0
male	284	100	95	100	189	100
**Age group (years)**						
≤20	40	14.1	13	13.7	27	14.3
21-30	90	31.7	34	35.8	56	29.6
31-40	64	22.5	17	17.9	47	24.9
41-50	51	18.0	20	21.1	31	16.4
51-60	29	10.2	9	9.5	20	10.6
>60	10	3.5	13	2.1	8	4.2
**Education**						
No Formal	64	22.5	21	22.1	43	23.4
Primary	69	24.3	28	29.5	41	22.3
Secondary	133	39.8	32	33.7	81	44
Tertiary	38	13.4	14	14.7	24	13
**Marital Status**						
Married	208	73.2	69	72.6	139	73.5
Unmarried	76	26.8	26	27.4	50	26.5
**Duration of work (Years)**						
<1	1	0.4	1	1.1	0	0
1-5	36	12.7	11	11.6	25	13.2
6-10	66	23.2	20	21.1	46	24.3
11-20	78	27.5	35	36.8	43	22.8
>20	103	36.3	28	29.5	75	39.7
**Occupational groups**						
Slaughterers	79	27.8	27	28.4	52	54.7
slaughterer/meat sellers	98	34.5	38	40	60	63.2
livestock seller/farmers	12	4.2	3	3.2	9	9.5
meat inspector/vets	20	7.0	8	8.4	12	12.6
meat sellers	66	23.2	20	21.1	46	48.4
Others	9	3.2	2	2.1	7	7.4

**Table 2:** ELISA antibody based distribution of brucellosis seroprevalence results

ELISA Test Results	Number of positive test samples	Percentage (N=95)
ELISA IgG alone	18	18.9
ELISA IgM alone	6	6.3
Both IgG and IgM antibodies	71	74.7
Negative for both ELISA tests	3	-
Total samples tested with ELISA=98	Total samples positive =95	

**Figure 1:**
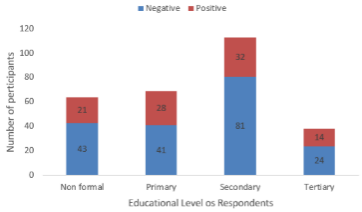
Seroprevalence of abattoir workers in Bauchi state based on educational levels

The Azare Abattoir recorded the highest (60%) seropositivity among participants sampled, and The Bauchi abattoir had the least (24.8%) seropositives. Age group 21-30 years accounted for the most seropositive participants 34(35.8%) by age grouping. The participants with the highest level of education (Tertiary education) had the least 14 (14.7%) participants seropositive (Figure 1). Seroprevalence was observed to increase steadily based on number of years of employment in an abattoir, from 1.1% in participants with less than one year of employment to 36.8% in participants with 11-20 years of employment in an abattoir. The slaughterer/meat seller group had the highest (40%) seropositivity recorded (Table 1). Significant risk factors included: slaughtering of animals in the abattoir with an odds ratio of 2.2 (1.2-3.7) and was significant with p value=0.003. People working in the abattoir while having an open cut or wound were twice [2.1 (1.1-3.9) p value=0.02] more likely to be infected with the pathogen. Other significant exposure observed include, eating while working at the abattoir [2.4 (1.1-4.90) p value= 0.02] and Assisting in animal parturition [2.3 (1.3-3.7) p value=0.001]. The use of Personal protective Equipment (PPE) like hand or footwear made workers half less likely to contract the disease [0.5 (0.3-0.9) p values 0.02 (Table 3). In logistic regression model following factors that were significant at bivariate analysis at a p < 0.05, slaughtering animals, working in the abattoir while having an open cut or wound, use of PPEs, eating while working in the abattoir and assisting in animal parturition were included in the model. Following stepwise elimination two factors remained statistically significant in the logistic regression model: participating in the slaughter of animals remained associated with seropositivity to human brucellosis (Adjusted Odds Ratio (AOR) = 1.92; CI 95% = 1.03-3.59, p = 0.04) and assisting in animal parturition (AOR = 2.43; CI 95% = 1.40 - 4.23, p = 0.002). All other factors had no statistical association with seropositivity to brucellosis among abattoir workers in this study (Table 4).

**Table 3:** Bivariate analysis of factors associated with brucellosis among abattoirs workers in Bauchi state

Variables	Number Tested	Seropositive N (%)	Seronegative N (%)	OR (95%CI)	P value
**Work exposure**					
Slaughtering of animals	169	68(40.2)	101(59.8)	2.2(1.2-3.7)	0.003*
Processing animal hide/skin	253	84(33.2)	169(66.8)	0.9(0.4-2.0)	0.80
Use of protective hand or foot wear (PPE)	115	29(25.2)	86(74.8)	0.5(0.3-0.9)	0.02*
Selling meat	162	57(31.1)	105(64.8)	1.20(0.7-1.98)	0.47
Working in abattoir while having open cut/wound	216	80(33)	136(66)	2.1(1.1-3.9)	0.02*
Collection of abattoir wastes/working as cleaner	189	84(35.2)	105(64.8)	1.2(0.7-2.0)	0.48
**Food Exposure**					
Eating while working in the abattoir	233	85(36.5)	148(63.5)	2.4(1.1-4.9)	0.02*
Drinking pasteurized milk	213	65(30.3)	148(69.4)	0.6(0.3-1.04)	0.07
Eating raw meat	43	14(32.6)	29(67.4)	0.95(0.5-1.9)	0.90
**Other Exposures**					
Assisting in animal parturition	145	61(42.1)	84(57.9)	2.3(1.3-3.7)	0.001*
Keeping animals in the house	184	66(35.9)	118(64.1)	1.4(0.8-2.3)	0.24
Milking animals/Processing Milk	28	14(50)	14(50)	2.16(0.98-4.7)	0.051

* Values significant at p<0.05

**Table 4:** Logistic regression model of significant variables in brucellosis among abattoirs workers in Bauchi state

Variables	Odds Ratio	95% CI	SE	Z-Statistics	P-value
Slaughtering animals	1.92	1.03-3.59	0.32	2.04	0.04*
Working in the abattoir while having open cut or wound	1.94	0.89-4.23	0.40	1.67	0.09
Use of protective hand or foot wear (PPE)	0.60	0.34-1.05	0.29	-1.80	0.07
Eating while working in the abattoir	0.39	0.21-0.73	0.32	-2.95	0.003
Assisting in animal parturition	2.43	1.40-4.23	0.28	3.15	0.002*

## Discussion

The findings from this study show that the seroprevalence of brucellosis among abattoir workers in Bauchi state is high. The Azare abattoir which has a livestock market situated close to it and attracted traders and animal owners from other communities from within and outside the state including Yobe and Kano state, had the most seropositive participants. The age groups most at risk was observed to be the younger age group of workers, these were mostly apprentices of much older butchers, they did most of the physical work requiring direct contact with the animal carcasses. Slaughtering of animals in northern Nigeria is a male dominated job. This is evident where all the workers that participated in the study were men. Among the various categories of abattoir workers that we screened, workers who participated in slaughtering animals and selling meat at the markets and stalls had the highest seropositivity rate. Factors associated with seropositivity include: slaughtering animals in the abattoirs, assisting in animal parturition, working in the abattoir while having an open cut or wound and eating while working in the abattoir. We also observed that workers who used PPEs like protective hand and foot wear while working in the abattoir were at least twice less likely to become seropositive with the brucella organism. This study found that seropositivity to the brucella organism increased with ascending number of years of work or employment in the abattoirs. Seropositivity was observed to increase steadily with increasing number of years of employment or service in the abattoir. Participants who have been employed for between 11-20 years and more than 20 years showed the highest numbers of seropositivity to the brucella organism.

The same was observed in another study carried out in 2010 in Abuja [15], where it was also observed that working in the abattoir for more than five years was a significant variable to seropositivity to human brucellosis. The age group 21-30 years had the most seropositive in the study, this may be owing to the fact that these set of people were involved in most tasks in the abattoirs that bring them in contact with animals, meat, blood and other infectious products. Equally the older workers who were more likely to be involved in supervisory tasks in the abattoir were observed to be less at risk of brucellosis. The findings in this study indicate that among the various occupational groups found in the abattoir, the butchers who slaughtered animals and also sold meat to customers at stalls and shops were most likely to be seropositive. This might be because these jobs carried a higher risk to physical injury from cuts and other work-related hazards and they had more contact with animals and animal tissue. Veterinarians and meat inspectors were observed to be less at risk and were more likely to use personal protective equipment (PPE). Studies in Ethiopia also revealed that because veterinarians and workers with post-secondary education background have considerable knowledge about the diseases during their professional training and also have good amount of practical knowledge through their experiences, they were less at risk of human brucellosis [17]. Bivariate, we found that worker involved in slaughtering animals at the abattoir were two times more likely to develop human brucellosis (p=0.003). This is consistent with the reports of studies done in Tanzania indicating significant risk among workers involved in cutting animal throats at the abattoirs [18].

People working in the abattoir while having an open cut or a wound were twice as likely to develop seropositivity to brucellosis (p=0.02). Inoculation through cuts and abrasions in the skin is one of the modes of transmission of brucellosis [6]. Other studies have reported similar findings where persons with bruises or cuts were more likely to be infected [15]. Workers assisting in animal parturition were found to be twice more likely to be seropositive for brucellosis (p=0.001). Farmers (and butchers) in Africa have been known to attempt assisting in parturition, handling retained placentas and trading in gravid uteruses [19]. Infected fetuses and placenta have been observed to carry high doses of infective organisms [6] direct contact with fetuses and uterine contents from livestock that have been shown to a significant risk in other studies [20,21] . Workers were observed to be less likely to be seropositive to brucellosis when they used Personal Protective Equipment (PPEs) like hand and foot wear (0.02) same was observed in other studies where use of PPEs was found to be a protective factor against brucellosis among slaughter house workers in Iran [22] and Tanzania [18]. In a study in Cameroon, all Brucella IgG seropositive respondents did not use of personal protective equipment (such as gloves) during work [21]. In another study it was reported that the constant use of full protective gear among few participants was most protective [23]. Although other studies associated eating raw or uncooked meat, unpasteurized milk and milk products with brucellosis [15,23] we found that there was a two-fold likelihood of seropositivity where participants admitted to eating food or drinks while working in the abattoir premises (p=0.02). This cross-sectional survey is limited by scope, (abattoir-based) infection/exposure cannot be said to be restricted to the abattoir and abattoir practices alone. There is the likelihood of infection form the community and the environment where participants work and live, especially the diverse nature of trade in livestock and transhumance system of animal husbandry practiced in Nigeria.

## Conclusion

Brucellosis is a public health problem among abattoir workers in Bauchi state, with high seroprevalence. The occupational risk practices of importance include slaughtering of animals, working without PPEs and working with uncovered skin abrasions, cut or wound. Other risk factors of brucellosis transmission among abattoir workers are; assisting in animal parturition. The analysis revealed that the disease can be prevented among abattoir workers through the use of personal protective devices during slaughter, advocating good general hygiene and training of personnel handling parturition. We recommend regular screening of abattoir workers and provide appropriate medical care to affected workers. The state Ministry of Agriculture should also organize regular health education forum for abattoir workers and other affected staff on the risks they face from brucellosis and other zoonotic occupational/ biorisks related to work. The Nigeria Centre for Disease Control should strengthen surveillance of zoonoses and explore means of strengthening zoonotic disease surveillance and incorporating some important zoonoses into current monitoring/surveillance system following the One Health Strategic Plan for prevention, detection and response to emerging and re-emerging diseases.

**Public health actions:** based on these findings, we conducted health talks for the abattoir workers in all study sites. Participants were enlightened about the causes, signs and prevention/control of brucellosis and this was done in collaboration with The State Ministry of Agriculture and Natural resources and The State Ministry of Health, Bauchi State. Seropositives were treated with the recommended full regimen multidrug therapy comprising Doxycycline tablets 200mg and Rifampicin Tablets 600 mg [6].

### What is known about this topic

- Brucellosis is a zoonotic disease that poses great risk to animal handlers and abattoir workers especially in sub-Saharan Africa;
- The disease can cause long term debilitating illness in human beings;
- The disease and its causal agents have been observed in the livestock population in Bauchi state, Nigeria.

### What this study adds

- There is a high seroprevalence (33.5%) of brucellosis among abattoir workers in Bauchi state, Nigeria;
- We identified slaughtering of animals and assisting in animal parturition as important risk factors of brucellosis among abattoir workers in Bauchi state, Nigeria;
- Proper use of personal protective equipment (PPE) was observed to reduce the risk of infection among abattoir workers.
